# Editorial: Triangle crosstalk: Gut microbiota, immune reaction and metabolism

**DOI:** 10.3389/fmicb.2023.1141016

**Published:** 2023-01-31

**Authors:** Cong-Qiu Chu, Yubin Luo, Yuan-Ping Han, Haitao Niu

**Affiliations:** ^1^Division of Arthritis and Rheumatic Diseases, Oregon Health and Science University, Portland, OR, United States; ^2^Innovent Biologics (USA), Rockville, MD, United States; ^3^Department of Rheumatology and Immunology, Rare Disease Center, Institute of Immunology and Inflammation, West China Hospital, Sichuan University, Chengdu, Sichuan, China; ^4^The College of Life Sciences, Sichuan University, Chengdu, Sichuan, China; ^5^Institute of Laboratory Animal Sciences, School of Medicine, Jinan University, Guangzhou, Guangdong, China

**Keywords:** microbiome, metabolites, immune response, crosstalk, dysbiosis

Gut microbiota exerts pleiotropic roles in human health and wellbeing, ranging from digestive function and absorption of nutrients, defense against infection, to the regulation of immune system development and immune homeostasis. Apart from the direct contact with the mucosal barrier of the host tissue, gut microbes are beneficial to the host through production of a variety of metabolites which may act on distant sites of the body. The concept of gut-other organ system axis, such as the “gut-lung axis” and “gut-joint axis” has been introduced to depict the relation of gut microbiota on the distant organ systems (Budden et al., 2017). Although there were many studies indicating the correlation between the gut microbiome, metabolites, and immune reaction, there is a huge knowledge gap as to how they interact and what are the outcomes.

The question is how to identify the key intestinal bacteria or metabolites, how to illustrate the crosstalk between microbiome, metabolites, and host immune reaction in disease, and how to take advantage of this triangle crosstalk in the future studies for clinical diagnosis and/or intervention. Four articles in this collection are concerning gut dysbiosis and metabolites in rheumatic diseases. Using untargeted metabolomics survey, Zhu et al. analyzed plasma metabolites of 244 patients with active disease (DAS28 5.5 ± 1.5) of rheumatoid arthritis (RA) and demonstrated that 63 metabolites are differently expressed compared with healthy controls. In particular, L-arginine and phosphorylcholine are substantially increased in RA patients. Not surprisingly, the changes in metabolites in RA patients were associated with dysbiosis of gut microbiota. Of particular interest is that increased L-arginine level was positively correlated with enriched fecal Rhodotorula of fungi. Several previous studies have shown alteration of fecal bacterial composition in RA patients but only few analyzed fungal composition (Lee et al., 2022; Sun et al., 2022). Another study analyzed fecal metabolites in RA patients and found depletion of L-arginine (Yu et al., 2021). These studies apparently have some common limitations: whether RA patients in the studies were treated with disease modifying anti-rheumatic drugs (DMARDs) was not clearly stated. Ideally, future studies should include the newly onset, DMARDs-naïve patients and focus on fungal composition concomitantly with analysis of fecal and blood metabolites to further delineate the changes of metabolites in correlation with fungal composition. In a systematic review of 31 studies investigating metabolites in spondyloarthritis (SpA) patients, Huang et al. (see also Huang et al., corrigendum) found that the profile of metabolites in SpA patients is distinctly different from that of healthy controls. In general, higher levels of metabolites of glucose, succinic acid, malic acid and lactate in carbohydrate metabolism, but lower levels of fatty acid in lipid metabolism; tryptophan and glutamine in amino acid metabolism were found in SpA patients. However, inconsistent results were also evident among studies which are likely due to the heterogeneous patient populations and various sources of samples ranging from plasma, synovial fluids, feces and urine. Interestingly, conventional DMARDS nor biological DMARDs treatment were able to normalize the changes of metabolites although they could control the clinical disease activity. In the narrative review, Wu et al. discussed the “gut-lung axis” focusing on interstitial lung fibrosis in the setting of a variety of lung conditions including idiopathic pulmonary fibrosis, fibrosis associated with systemic sclerosis, radiation induced lung fibrosis and silicosis-related pulmonary fibrosis. Changes of gut microbiota are seen in each of the conditions or their animal models. Discussion extended to mechanism how these metabolites affect immune cell function and fibroblasts and myofibroblasts. In a preliminary study, Li M. et al. reported the distinctive profile of gut microbiota in patients with non-infective anterior scleritis than that of patients associated with RA. Further experiments are required to investigate how different groups of microbes might affect the inflammation of eyes.

Metabolic syndrome affects about 25% of the population worldwide and the prevalence is increasing (Saklayen, 2018). Gamma glutamyl transpeptidase (GGT) is a biomarker for hepatocyte necrosis and surrogate marker for subclinical inflammation and independent risk factor for atherosclerosis. Individuals with metabolic syndrome having elevated GGT levels may render an increased risk for cardiovascular disease. Sheng et al. found that men with metabolic syndrome accompanied with elevated GGT harbor more “harmful gut microbes” compared those without elevated levels of GGT. Importantly, individuals with elevated GGT levels can be recognized through routine health checkout and intervention to reduce the “harmful gut microbes” may be implemented early for a better prognosis.

In the three original research articles, investigators focused on manipulations to affect gut microbiome to change the clinical outcomes in animal models. In a rat liver transplant model, Yuan et al. transferred heme oxygenase-1 (HO-1) gene transfected bone marrow mesenchymal stem cells (HO-1/BMMSCs) into steatotic liver recipient rats. Surprisingly, HO-1/BMMSCs enhanced levels of gut *Desulfovibrionaceae* which promote energy metabolism, especially lipid metabolism, and increased the levels of butyrate-producing bacteria, such as *Lachnospiraceae*. The changes of these gut bacterial composition contribute to the improved steatosis and improved the survival of the recipients. These results exemplified the “gut-liver axis.” Jin et al. investigated the mechanism of action of two weight loss drugs, orlistat and ezetimibe by analyzing the effect of them on gut microbiome in animal models of high fat diet induced obesity. Orlistat significantly reduced the number of gut *Firmicutes, Alistipes*, and *Desulfovibrio* and simultaneously increased *Verrucomicrobia* and *Akkermansia*. In comparison, ezetimibe decreased *Proteobacteria* and *Desulfovibrio*, but increased *Bacteroides*. Apparently, orlistat and ezetimibe influenced different populations of gut bacteria although both can alleviate fat-diet induced obesity. Another group of investigators (Li Y. et al.) fed weaned piglets with different protein diets in their early life to observe the influence on growth of the animals. Three groups of animals were fed with 16% of proteins from fermented soybean (FSB) meal, fish meal (FM) or mixture of fish and soybean meal (MFSM), respectively. MFSM diet significantly improved growth performance of the animals. The effect was attributed to gut microbiome shaped by MFSM diet. Findings of these animal models have implications for human health and intervention of disease by manipulation of gut microbiome.

“抛砖引玉 (Casting a stone to attract jades)”—this Chinese saying reflects the intent of the topic editors. Research in some articles of this collection is preliminary and descriptive but has raised more questions to be answered. We hope investigators will be inspired for a deep dive into the insight of interaction between microbiome and host.

## Author contributions

All authors contributed to the manuscript drafting and revision. All authors contributed to the article and approved the submitted version.
